# A Sensitive and Simple HPLC-UV Method for Trace Level Quantification of Ethyl *p*-Toluenesulfonate and Methyl *p*-Toluenesulfonate, Two Potential Genotoxins in Active Pharmaceutical Ingredients

**DOI:** 10.3797/scipharm.1106-23

**Published:** 2011-09-20

**Authors:** Amasa Nageswari, Kallam Venkata Siva Rama Krishna Reddy, Khagga Mukkanti

**Affiliations:** 1 Center of Pharmaceutical Sciences, IST, Jawaharlal Nehru Technological University, 500072, Kukatpally, Hyderabad, India; 2 Aptuit Laurus Private Limited, ICICI knowledge park, 500078, Turkapally, Hyderabad, India

**Keywords:** Threshold of toxicological concern, Genotoxin, Daily dosage, Active Pharmaceutical Ingredient, Validation

## Abstract

A sensitive and simple HPLC/UV method has been developed and validated for the determination of two potential genotoxic impurities, namely methyl *p*-toluenesulfonate (MPTS) and ethyl *p*-toluenesulfonate (EPTS) at trace levels in Pemetrexed sodium API. Applying the concept of threshold of toxicological concern (TTC), a limit of 3 ppm each for both genotoxins was calculated based on the maximum daily dose of API. A reversed phase LC method using UV detection was developed and validated. The method was found to be specific and selective for the application. The limit of detection (LOD) and limit of quantitation (LOQ) for both MPTS and EPTS was found to be 0.15 ppm (0.009 μg mL^−1^) and 0.5 ppm (0.03 μg mL^−1^), respectively, with respect to sample concentration. The calibration curves of MPTS and EPTS were linear over the concentration range from LOQ to 6 μg/mL. The method was found to be specific, precise, linear and accurate and has been successfully applied to determine the two genotoxins in commercial batches of the API.

## Introduction

**Fig. 1:**
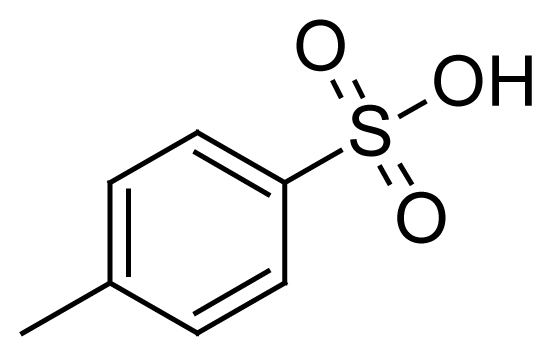
*p*-Toluenesulfonic acid

**Fig. 2:**
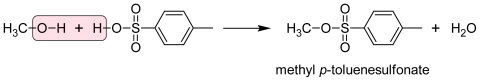
Formation of methyl *p*-toluenesulfonate

**Fig. 3:**
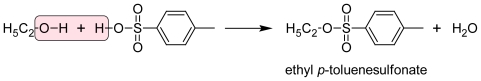
Formation of ethyl *p*-toluenesulfonate

*p-Toluenesulfonic* acid (pTSA) (Figure 1), a strong organic acid, is a common reagent used in pharmaceutical industry as an “organic-soluble” acid catalyst or in purification steps of chemical synthesis of a drug substance. The applications of p-TSA include but are not limited to: Acetalization of an aldehyde [1], Esterification of carboxylic acids [2], and Transesterification of an ester [3]. The presence of any residual alcohols like methanol and ethanol from synthetic reaction or recrystalization steps may result in the formation of corresponding alkyl tosylates, which are known to be Potential Genotoxic Impurities (PGIs) [4, 5]. These PGIs, alkyl tosylates due to their DNA alkylation action can induce mutagenic, carcinogenic and teratogenic effects [6]. The manufacturing process of the subject API involves the solvents methanol and ethanol and p-TSA as a reagent for salt formation. Hence there is a highly likelihood for the formation of the two genotoxins, methyl *p*-tolulenesulfonate (Figure 2), and ethyl *p*-toluenesulfonate (Figure 3) which cannot be ignored in the API.

The assessment and control of Genotoxic impurities (GTIs) in pharmaceutical products has received considerable attention in recent years. The International Conference on Harmonization (ICH), section Q3A, provides guidance on impurities in New Drug Substances [7]. It states that lower reporting thresholds can be appropriate if the impurity is unusually toxic. The European Medices Agency (EMEA) issued guidelines for GTI limits and included the concept of threshold toxicological concern (TTC) to define acceptable risk for the new active substances [8]. Recently, in 2008 US FDA (United States Food and Drug Administration) has also come up with the draft guidelines on genotoxic and carcinogenic impurities in drug substances and drug products [9]. Pharmaceutical Genotoxic impurities may potentially increase the risk of cancer in patients. These guidelines describe ways to characterize, monitor and control the level of GTIs in drug substances and drug products. A maximum daily exposure target of 1.5 μg per day (acceptable Threshold of Toxicological Concern, TTC) is recommended in these guidelines [8–10].

Based on maximum daily dosage of selected drug substance used in this study, MPTS and EPTS are required to be controlled at a combined limit of 0.18 μg/mL in API.

Due to the increasing concern from the regulatory agencies with respect to potential genotoxic impurities, a number of sophisticated analytical techniques, namely Gas chromatographic methods utilizing both FID/MS detectors and HPLC/UPLC coupled with UV/MS detectors, are available for the estimation of Genotoxins in drug substances and drug products.

However, some of the analytical methods described in the literature for the determination of low levels of these Genotoxins define using HPLC-ECD with Boron-Doped Diamond Electrochemical Cell [11]. These methods are complicated and time-consuming when compared to the method proposed in the present study wherein a simple method is applied using HPLC technique with UV detection.

This paper presents a simple and sensitive HPLC method using UV detector that can accurately quantify both MPTS and EPTS at trace levels. The method was successfully applied to demonstrate that these genotoxins are controlled below TTC levels in the bulk drug and also can be applied for the estimation of MPTS and EPTS in other drug substances where maximum daily dosage is up to 2 g.

## Experimental

### Chemicals

Samples of ethyl *p*-toluenesulfonate and methyl *p*-toluenesulfonate were purchased from Sigma Aldrich Co., St.Louis, USA. The Drug substance, Pemetrexed disodium used for the study was received from Aptuit Laurus Private Limited, Hyderabad, INDIA. HPLC grade Acetonitrile, Methanol and AR grade Orthophosphoric acid were purchased from Merck, Darmstadt, Germany. High purity water was collected using Millipore Milli-Q plus water purification system.

### Instrumentation

The LC systems, used in the present study are Waters Alliance equipped with a Waters 2695 quaternary gradient pump, auto injector with 2489 UV Visible detector and Schimadzu LC-2010C HT equipped with quaternary gradient pump, auto injector UV visible detector. The output signal of both LC systems was monitored and processed through Empower 2 networking software; version build number 2154.

### Chromatographic conditions

The chromatographic separation was performed on a 5 μm particles, “Inertsil ODS-3V” (250 mm × 4.6 mm), column procured from GL science inc., Tokyo, Japan. The mobile phase consisted of 0.1% phosphoric acid in water and Acetonitrile in a ratio of 1:1 (v/v). The mobile phase was pre-mixed, filtered through a 0.45 μm nylon filter and degassed. The flow rate of the mobile phase was kept at 2.0 mL min^−1^. The temperature of column was maintained at 27°C and the detection was carried out at a wavelength of 225 nm. Injection volume was 20 μL and run time was 15 min. Samples were prepared in Methanol.

### Preparation of solutions

An Impurity stock solution of ethyl *p*-toluenesulfonate and methyl *p*-toluenesulfonate (0.6mg mL^−1^) was prepared by dissolving appropriate amount in methanol. Test solutions of drug substance 60 mg mL^−1^ were prepared by dissolving appropriate amount in methanol.

Sample solutions of three different lots of drug substance were prepared in triplicate at a concentration of 60 mg mL^−1^ in Methanol by dissolving appropriate amount to perform the analysis.

### Analytical Method Validation

The developed chromatographic method was validated for specificity, sensitivity (limit of detection and limit of quantification), precision (repeatability, reproducibility and intermediate precision), linearity, range, accuracy and solution stability.

#### Specificity

The specificity of the optimized method was performed by injecting stock solutions of individual impurities to check resolution among MPTS, EPTS, methanol and drug substance under the same conditions mentioned under chromatographic conditions.

#### Sensitivity

Sensitivity of the method was proven by establishing the limit of detection (LOD) and limit of quantitation (LOQ) for MPTS and EPTS with signal-to-noise-ratios of 3:1 and 10:1, respectively. LOD and LOQ were determined by injecting a series of diluted solutions having known concentrations of impurities. Precision of the method was also carried out at the LOQ level by injecting six individual preparations of MPTS and EPTS at LOQ concentration by calculating %RSD for the areas of each peak.

#### Precision

Precision was determined through repeatability (intra-day) and intermediate (inter-day) precision. Precision of the method was checked by injecting six individual preparations of MPTS and EPTS at 0.18 μg/mL level for each impurity with respect to concentration of the drug substance. %RSD for content of each impurity was calculated.

Intermediate precision (ruggedness) of the method was evaluated by using three different columns, three different instruments on different days in the same laboratory at four different levels i.e. LOQ, 50%, 100% and 150% of the specification limit.

#### Linearity and Range

To establish the Linearity of the method, calibration solutions were prepared by diluting the impurity stock solution to the required concentrations at six different levels ranging from LOQ to 6 μg mL^−1^ of each impurity. The linearity graph was drawn with concentration of linearity solution on x-axis and mean area counts on y-axis. The slope, y-intercept, y-bias and correlation coefficient of the calibration curve were calculated.

#### Accuracy

To determine accuracy of the method, a recovery study was carried out by analyzing the drug substance spiked with impurities. Known amount of impurities were spiked to the previously analyzed samples at different concentration levels of LOQ, 50%, 100% and 150% at the specification limit of the drug substance concentration (60 mg mL^−1^). Each concentration level was prepared in triplicate. Percentage recoveries for MPTS and EPTS were calculated.

#### Solution stability

Solution stability of impurities standard (0.18 μg mL^−1^) was carried out by keeping them in tightly capped volumetric flasks at room temperature for 12 h. The solutions were analyzed at 1 h intervals up to 12 h.

## Results and Discussion

### LC Method optimization

The specification limit for genotoxic impurities (MPTS and EPTS) in the selected drug substance has been calculated based on TTC and maximum daily dose of drug substance i.e. 500 mg.

A maximum daily exposure target of Genotoxic impurities is 1.5 μg per day per person.

Hence specification limit for impurities in ppm = 1.5 μg/Day of the drug substance in g. i.e. 1.5/0.5 = 3.0 ppm.

The aim of the current study was to develop a specific and sensitive analytical method, which could not only separate both MPTS and EPTS from the drug substance but also could quantify the impurities at less than 50% to the specification limit.

The desired specificity of the method was achieved on a 5 μm particle size, “Inertsil ODS-3V” (250 × 4.6 mm) column with 0.1% ortho phosphoric acid in water and Acetonitrile (1:1 v/v) as mobile phase. Impurities were monitored by using UV Vis detector at 225 nm. Flow rate was 2.0 mL^−1^ and column temperature was 27 °C. Run time of the method was 15 min.

### Analytical parameters and validation

The developed method was validated to demonstrate that it is suitable for its intended use as per ICH guidelines [13, 14] (sensitivity, precision, accuracy, linearity, range, selectivity, and solution stability).

#### Sensitivity (Limit of detection and Limit of quantification)

**Fig. 4:**
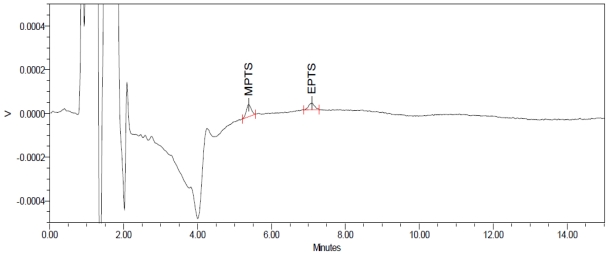
LOD (0.009 μg mL^−1^) chromatogram of methyl and ethyl *p*-toluenesulfonate

**Fig. 5:**
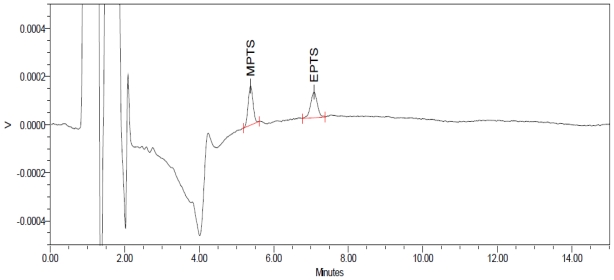
LOQ (0.03 μg mL^−1^) chromatogram of methyl and ethyl *p*-toluenesulfonate

Sensitivity was determined by establishing limit of detection (LOD) and limit of quantification (LOQ) for GTIs, which were estimated through signal to-noise ratio of 3:1 and 10:1, respectively, by injecting a series of dilute solutions having a known concentration. LOD of the impurity is defined as the lowest concentration that can be detected. LOD for MPTS and EPTS was found to be 0.009 μg mL^−1^ (Figure 4). LOQ is the lowest concentration of an impurity that can be quantified with acceptable precision and accuracy. LOQ for MPTS and EPTS was found to be 0.03 μg mL^−1^ (Figure 5). Precision study was also carried out at LOQ level by injecting six individual preparations of MPTS and EPTS by calculating the %RSD for the content in μg mL^−1^ of each impurity. Results are shown in Table 1.

#### Precision

Precision of the method was checked by injecting six individual preparations of both MPTS and EPTS impurities at four different levels i.e. LOQ, 50%, 100% and 150% of the specification limit. %RSD was calculated for the content of MPTS and EPTS in μg mL^−1^. Intermediate precision of the method was also evaluated using three different columns, three different instruments on different days, in the same laboratory. Results are summarized in Tables 1–3. Results of inter day precision generated on three different days were also calculated in terms of %RSD and the results are listed in Table 4.

#### Linearity

Linearity of the method was demonstrated using a six point calibration plot at concentrations ranging from LOQ to 100 ppm with respect to sample concentration (i.e. 0.03, 0.18, 0.6, 1.2, 3 and 6 μg mL^−1^). The correlation coefficient of linear regression is greater than 0.999 for each impurity.

#### Accuracy

**Fig. 6:**
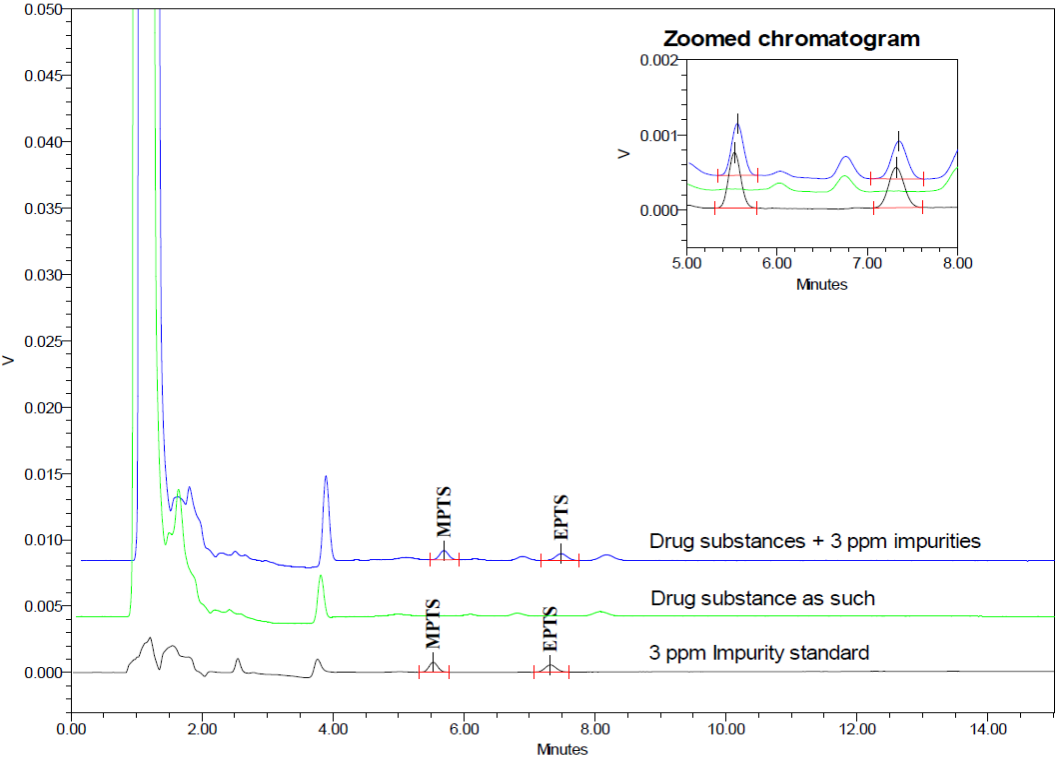
Chromatogram of Accuracy at 100% specification level (0.18 μg mL^−1^)

Accuracy of the method was evaluated at four concentration levels in triplicate. Analysis was carried out by spiking the impurities to the drug substance at different concentrations of LOQ, 50%, 100% and 150% of specification limit of MPTS and EPTS (i.e. 0.03, 0.09, 0.18 and 0.27 μg mL^−1^). The % recoveries were calculated for both GTIs which were found to be 90 to 99 % and 89 to 103% for MPTS and EPTS, respectively. Typical overlaid chromatogram of drug substance and drug substance spiked with impurities is shown in Figure 6. Results of accuracy study are summarized in Tables 5–7.

#### Specificity

**Fig. 7:**
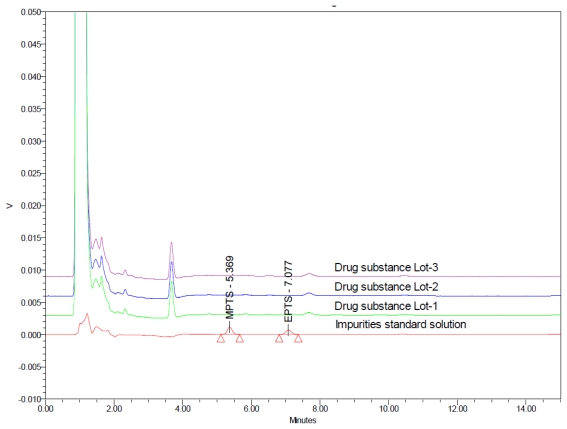
Overlaid chromatogram of different lots of drug substance with standard solution of 0.18 μg mL^−1^ of MPTS and EPTS impurities

Selectivity of the method for the estimation of MPTS and EPTS in drug substance described in the present study was proven by injecting separately solutions of drug substance, other related impurities that are expected to be present in the drug substance and the two Genotoxins where drug substance and its related impurities eluted below 4 minutes and GTIs eluted after 5 min (Figure 7).

#### Solution stability

Solution stability of impurities was studied up to 12 h at room temperature and found no significant difference in the area of impurities was observed from initial to 12 h period. P value calculated using student’s t-test was 0.10 (10%) [15]. The similarity factors for both MPTS and EPTS in solution stability experiments were 1.00 and 1.03, respectively.

#### Batch analysis

Three different lots of Pemetrexed disodium drug substance were analyzed in triplicate in the validated method for estimation of MPTS and EPTS and found that both the impurities were below Detection Limit (< 0.009 μg mL^−1^).

## Conclusion

The present study describes a sensitive, simple, rapid and accurate validated LC method by using UV detection for estimation of potential GTIs, MPTS and EPTS in the drug substance. The method was found to be selective, sensitive, precise, accurate and linear for the determination of GTIs. This method is suitable for quantification of MPTS and EPTS in any other drug substances with a maximum daily dose up to 2.0 g.

## Figures and Tables

**Tab. 1:** Intraday Precision at four levels from LOQ to 150% of specification limit in Day-1

#	Concentration with respect to specification limit (%)	%RSD of MPTS content in μg mL^−1^	%RSD of EPTS content in μg mL^−1^
1	LOQ	1.4	0.8
2	50%	0.4	0.6
3	100%	1.3	1.9
4	150%	0.3	0.4

n= 6 preparations.

**Tab. 2:** Intraday Precision at four levels from LOQ to 150% of specification limit in Day-2

#	Concentration with respect to specification limit (%)	%RSD of MPTS content in μg mL^−1^	%RSD of EPTS content in μg mL^−1^
1	LOQ	1.5	0.4
2	50%	0.6	1.6
3	100%	0.4	0.5
4	150%	0.3	1.5

n= 6 preparations.

**Tab. 3:** Intraday Precision at four levels from LOQ to 150% of specification limit in Day-3

#	Concentration with respect to specification limit (%)	%RSD of MPTS content in μg mL^−1^	%RSD of EPTS content in μg mL^−1^
1	LOQ	0.9	1.5
2	50%	1.2	1.5
3	100%	0.6	1.1
4	150%	0.4	1.9

n= 6 preparations.

**Tab. 4:** Interday Precision of three different days at four levels from LOQ to 150% of specification limit

#	Concentration with respect to specification limit (%)	%RSD of MPTS content in μg mL^−1^	%RSD of EPTS content in μg mL^−1^
1	LOQ	9.4	7.7
2	50%	8.0	9.3
3	100%	8.4	2.9
4	150%	8.1	9.1

n= 6 preparations.

**Tab. 5:** Accuracy at four levels from LOQ to 150% to the specification limit in Day-1

#	Concentration to specification level	MPTS spiked to API*			EPTS spiked to API*		

		X (μg mL^−1^)	Y (μg mL^−1^)	%Mean recovery ±SD	X (μg mL^−1^)	Y (μg mL^−1^)	%Mean recovery ±SD
1	LOQ	0.0307	0.0290	94.5±1.19	0.0308	0.0304	98.6±1.93
2	50%	0.0968	0.0968	94.6±0.85	0.0896	0.0919	102.5±0.61
3	100%	0.1839	0.1671	90.8±0.38	0.1845	0.1680	91.1±0.67
4	150%	0.2903	0.2784	95.9±0.52	0.2687	0.2750	102.4±0.49

n= 3 determinations; X…amount spiked; Y…amount recovered.

**Tab. 6:** Accuracy at four levels from LOQ to 150% in Day-2

#	Concentration to specification level	MPTS spiked to API*			EPTS spiked to API*		

		X (μg mL^−1^)	Y (μg mL^−1^)	%Mean recovery ±SD	X (μg mL^−1^)	Y (μg mL^−1^)	%Mean recovery ±SD
1	LOQ	0.0305	0.0286	93.8±1.00	0.0332	0.0295	89.0±0.35
2	50%	0.0923	0.0836	90.6±0.06	0.0947	0.0886	93.6±0.36
3	100%	0.1826	0.1815	99.3±0.70	0.1791	0.1830	102.2±0.95
4	150%	0.2770	0.2627	94.8±1.60	0.2840	0.2749	96.8±0.50

n= 3 determinations; X…amount spiked; Y…amount recovered.

**Tab. 7:** Accuracy at four levels from LOQ to 150% in Day-3

#	Concentration to specification level	MPTS spiked to API*			EPTS spiked to API*		

		X (μg mL^−1^)	Y (μg mL^−1^)	%Mean recovery ±SD	X (μg mL^−1^)	Y (μg mL^−1^)	%Mean recovery ±SD
1	LOQ	0.0307	0.0290	94.5±1.19	0.0332	0.0295	89.0±0.35
2	50%	0.0953	0.0943	98.9±0.42	0.0893	0.0825	92.4±0.15
3	100%	0.1839	0.1671	90.8±0.38	0.1791	0.1830	102.2±0.95
4	150%	0.2858	0.2559	89.6±0.72	0.2680	0.2682	100.1±0.40

n= 3 determinations; X…amount spiked; Y…amount recovered.
